# Supplementary material for: Treacle controls the nucleolar response to rDNA breaks via TOPBP1 recruitment and ATR activation
Source: Nat Commun. 2020 Jan 8;11:123. doi: 10.1038/s41467-019-13981-x (PMC6949271; doi:10.1038/s41467-019-13981-x)
Supplement: Supplementary file 1 — Supplementary Information [file 41467_2019_13981_MOESM1_ESM.pdf]

Treacle controls the nucleolar response to rDNA breaks via TOPBP1 recruitment and ATR activation

Mooser et al.

Supplementary Information

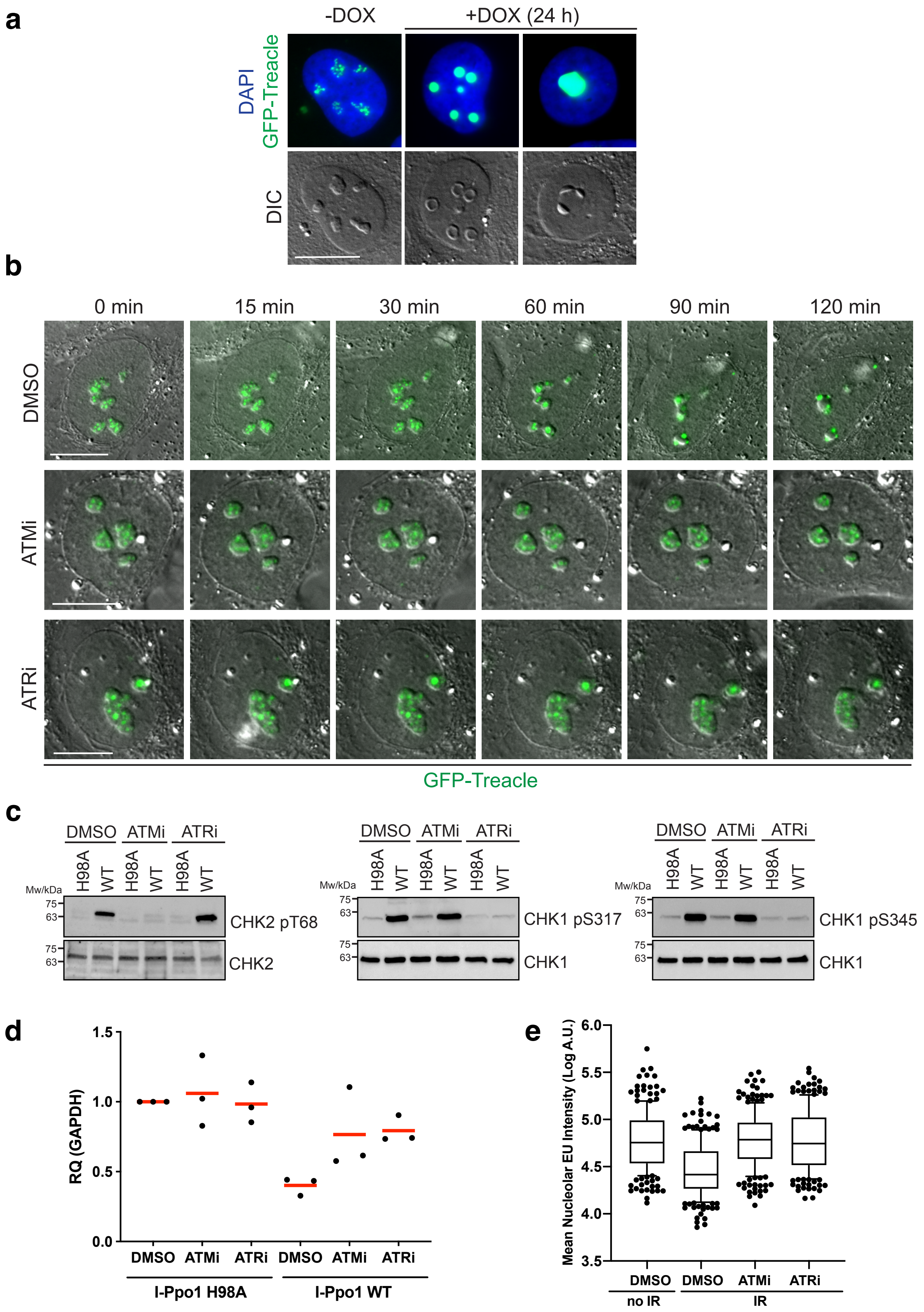

Supplementary Figure 1: ATM and ATR dependent nucleolar segregation, CHK1/CHK2 phosphorylation and Pol I inhibition. **a** Stable expression of a Tetracycline-inducible GFP-Treacle expression cassette along with the Tetracycline repressor allowed for low levels of GFP-Treacle expression in the absence of induction by Doxycicline (-DOX). Note that the addition of Doxycycline to the medium for 24 h leads to destruction of the nucleolar structure due to Treacle overexpression. **b** Time-lapse microscopy of U2OS cells expressing low levels of GFP-tagged Treacle after transfection with I-Ppo1 and after pre-treatment with DMSO, ATMi and ATRi, respectively. **c** Western blots of total cell extracts of cells transfected with I-Ppo1 wild type (WT) and catalytic mutant H98A. **d** qRT-PCR of rRNA levels after I-Ppo1 transfection in control, ATMi and ATRi treated cells (red bars represent the man; n=3). **e** Quantification of nucleolar EU incorporation before (n=204) and after 5 Gy of IR in control (DMSO, n=196), ATMi (KU-55933, n=206) and ATRi (VE-821, n=196) treated cells (boxes represent the median with 25-75 percentile range and whiskers represent the 5-95 percentile range. Data points outside of this range are shown individually). All scalebars = 10 μm.

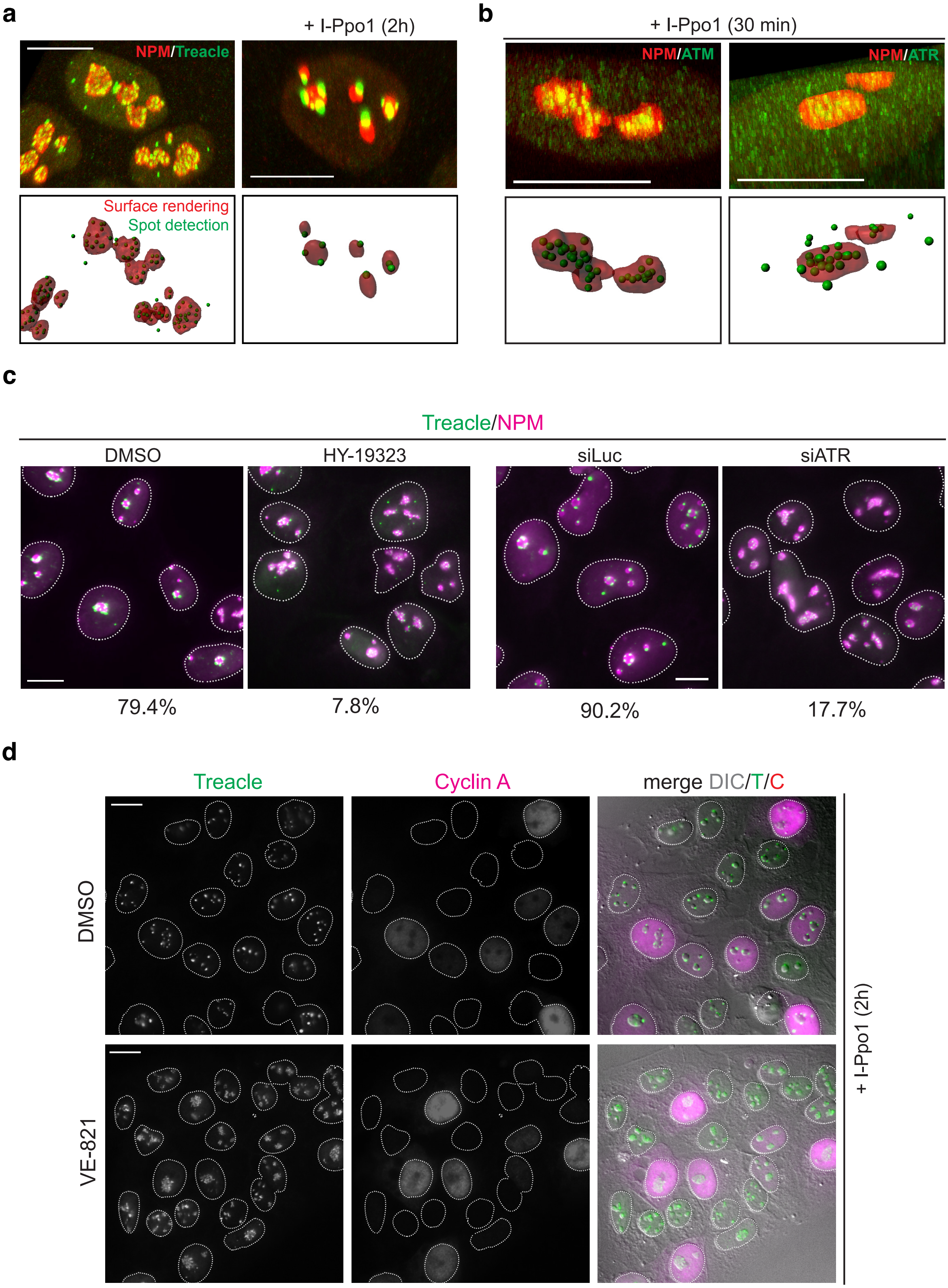

Supplementary Figure 2: ATM and ATR nucleolar recruitment and cell cycle analysis. **a** Example of nucleolar surface rendered 3D reconstruction of confocal z-stacks by IMARIS. **b** 3D reconstruction by IMARIS reveales recruitment of ATM and ATR in the nucleoli 30 min after I-Ppo1 transfection. **c** Defective nucleolar segregation in cells treated with the ATR inhibitor HY-19323 and in cells depleted of endogenous ATR by siRNA. **d** ATR inhibitors block nucleolar segregation throughout the cell cycle, even in Cyclin A negative (G1) cells. All scalebars = 10 μm.

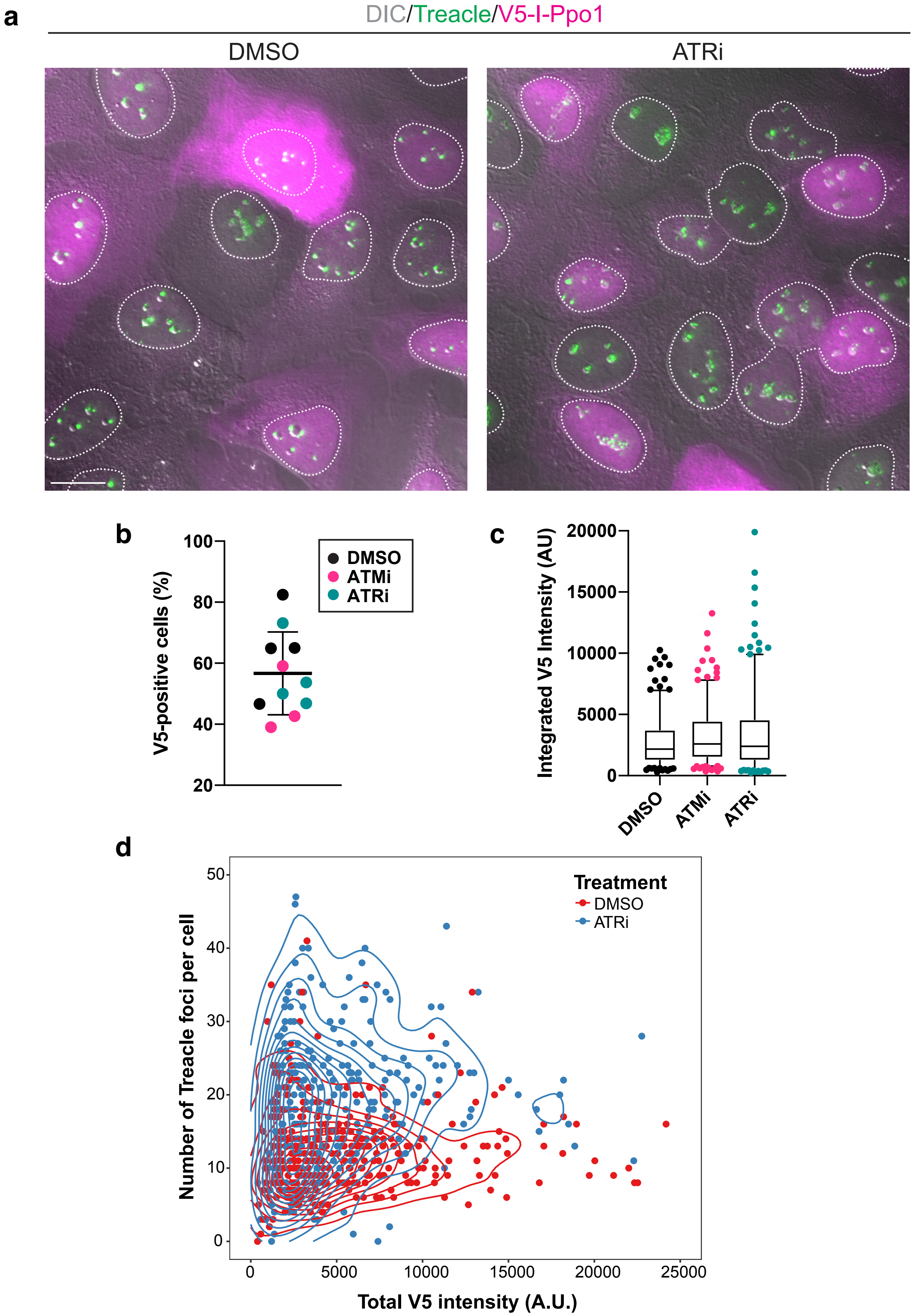

Supplementary Figure 3: I-Ppo1 expression is not affected by ATM and ATR inhibitors. **a** Immunofluorescence staining with Treacle and V5 antibodies indicates that very low V5-IPpo1 expression levels lead to nucleolar segregation. **b** Quantification of the fraction of V5-I-Ppo1 positive cells in 11 independent experiments. Color of individual datapoints indicate treatment. Bar represents mean and error bars SD (n=11). **c** Quantification of V5-I-Ppo1 staining intensity per cell nucleus in DMSO (n=224), ATMi (n=246) and ATRi (n=245) treated cells (boxes represent the median with 25-75 percentile range and whiskers represent the 5-95 percentile range. Data points outside of this range are shown individually). **d** Scatter blot with 2D kernel density estimates showing the number of Treacle foci per cell in relation to V5-I-Ppo1 staining intensity. Note that very low V5-I-Ppo1 expression levels induce nucleolar segregation (as evidenced by a significant reduction in the number of Treacle foci per cell nucleus in DMSO treated cells). All scalebars = 10 μm.

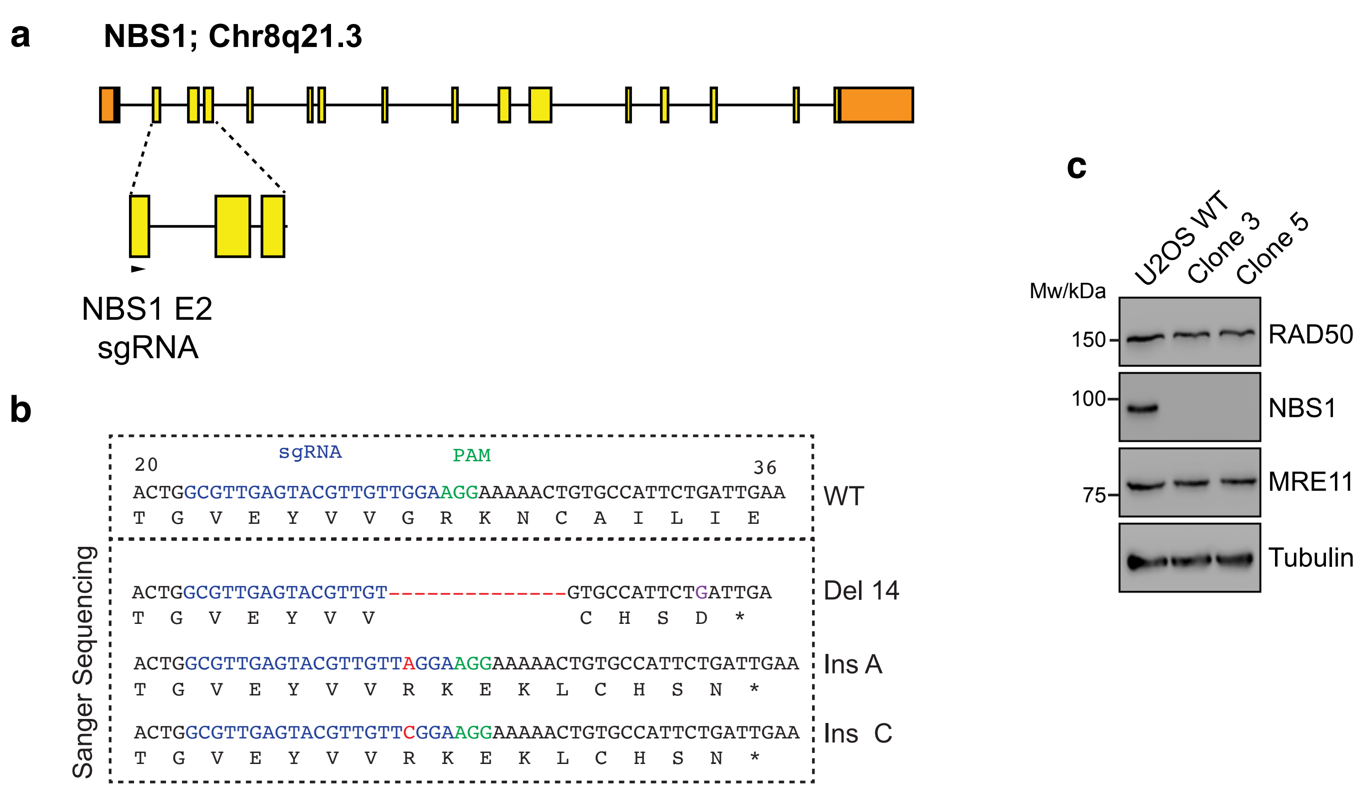

Supplementary Figure 4: Generation of NBS1 knock-out U2OS cells. **a** Schematic representation of the human NBS1 gene locus, illustrating the hybridization site of the gRNA selected for the generation of ΔNBS1 cell line used in this study **b** Sanger sequencing revealed indel mutations in all three NBS1 alleles present in ΔNBS1 clone 5. **c** Western blot of extracts derived from U2OS WT and two ΔNBS1 clonal cell lines.

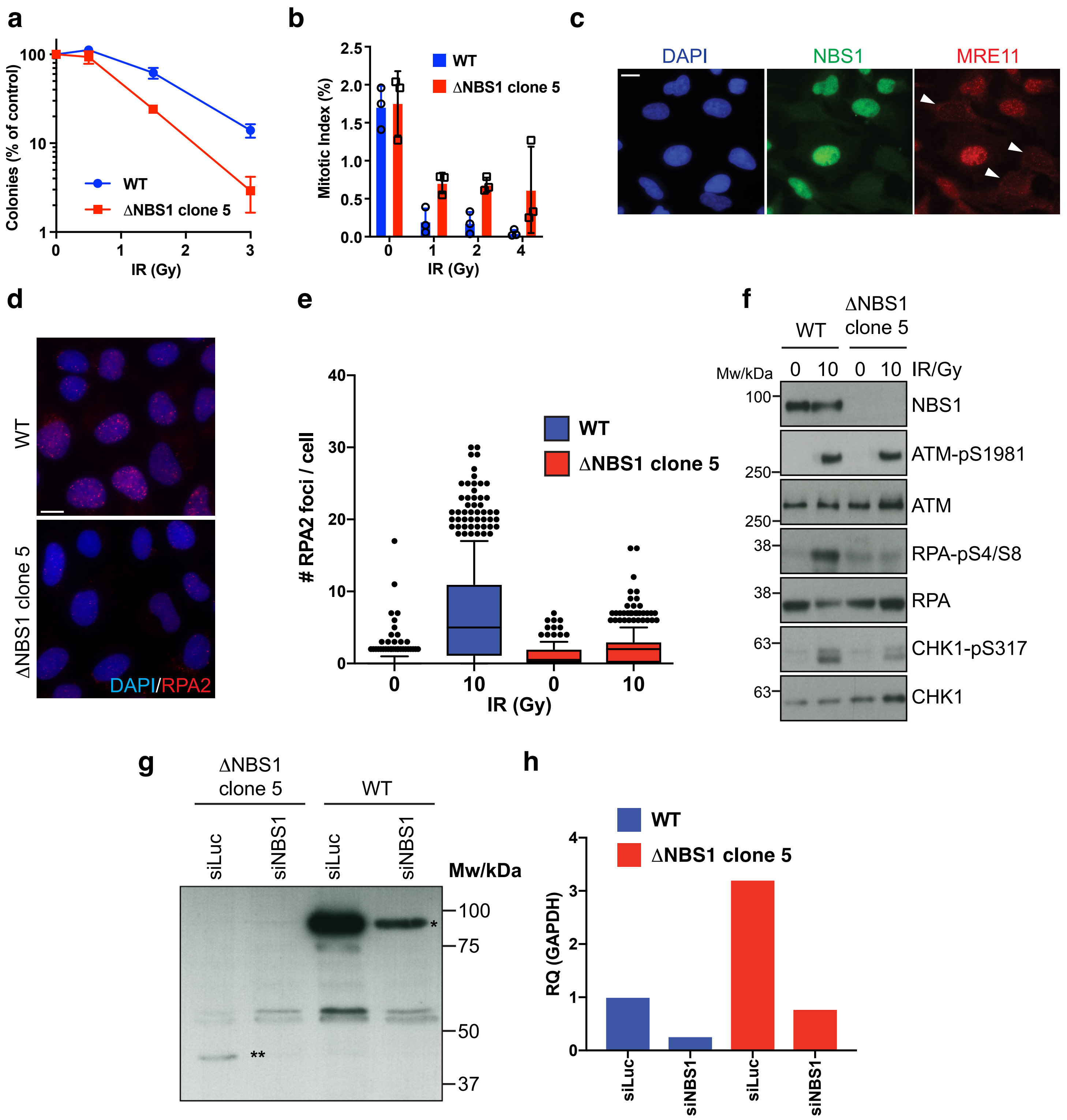

Supplementary Figure 5: Characterization of NBS1 knock-out cell lines. **a** Clonogenic survival assay of IR-treated NBS1 deficient clone 5 versus control cells (points represent mean of three biological replicates, error bars represent standard deviation; n=3) **b** G2/M checkpoint assay of IR-treated NBS1 deficient clone 5 versus control cells (bars represent mean of three biological replicates, error bars represent standard deviation; n=3). **c** Immunofluorescence of a 1:1 mixture of NBS1 deficient clone 5 cells and control cells. **d** DNA end resection as monitored by RPA2 foci formation 3h after 3 Gy of IR in NBS1 deficient clone 5 versus control cells. **e** Quantification of the experiment in **e** (boxes represent the 25-75 percentile range and whiskers represent the 5-95 percentile range. Data points outside of this range are shown individually). **f** Western blots of total cell extract of U2OS WT cells and NBS1 deficient clone 5 cells after 10 Gy of IR. **g** Western blot of total cell extracts prepared from NBS1 siRNA treated NBS1 deficient clone 5 and control cells. ** indicates a siRNA-sensitive 40 kDa band present only in the NBS1 deficient clones. **h** qRT-PCR of NBS1 mRNA prepared from NBS1 siRNA treated NBS1 deficient clone 5 and control cells. All scalebars = 10 μm.

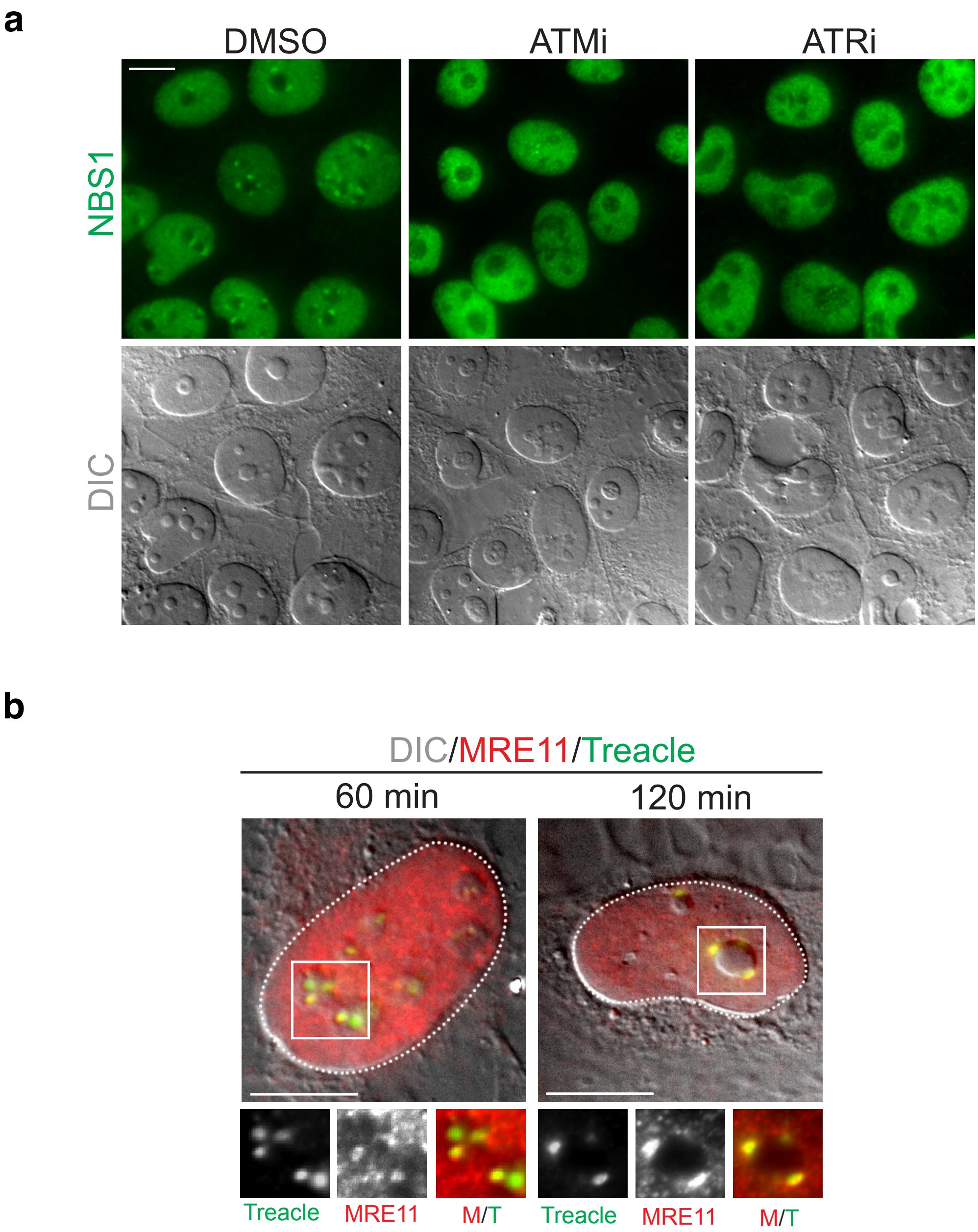

Supplementary Figure 6: NBS1 and MRE11 nucleolar recruitment in response to rDNA breaks. **a** NBS1 localization 2 h after I-Ppo1 mRNA transfection and after pre-treatment of cells with DMSO, ATMi and ATRi, respectively. **b** Timecourse of MRE11 nucleolar recruitment and co-localization with Treacle after I-Ppo1 expression. All scalebars = 10 μm.

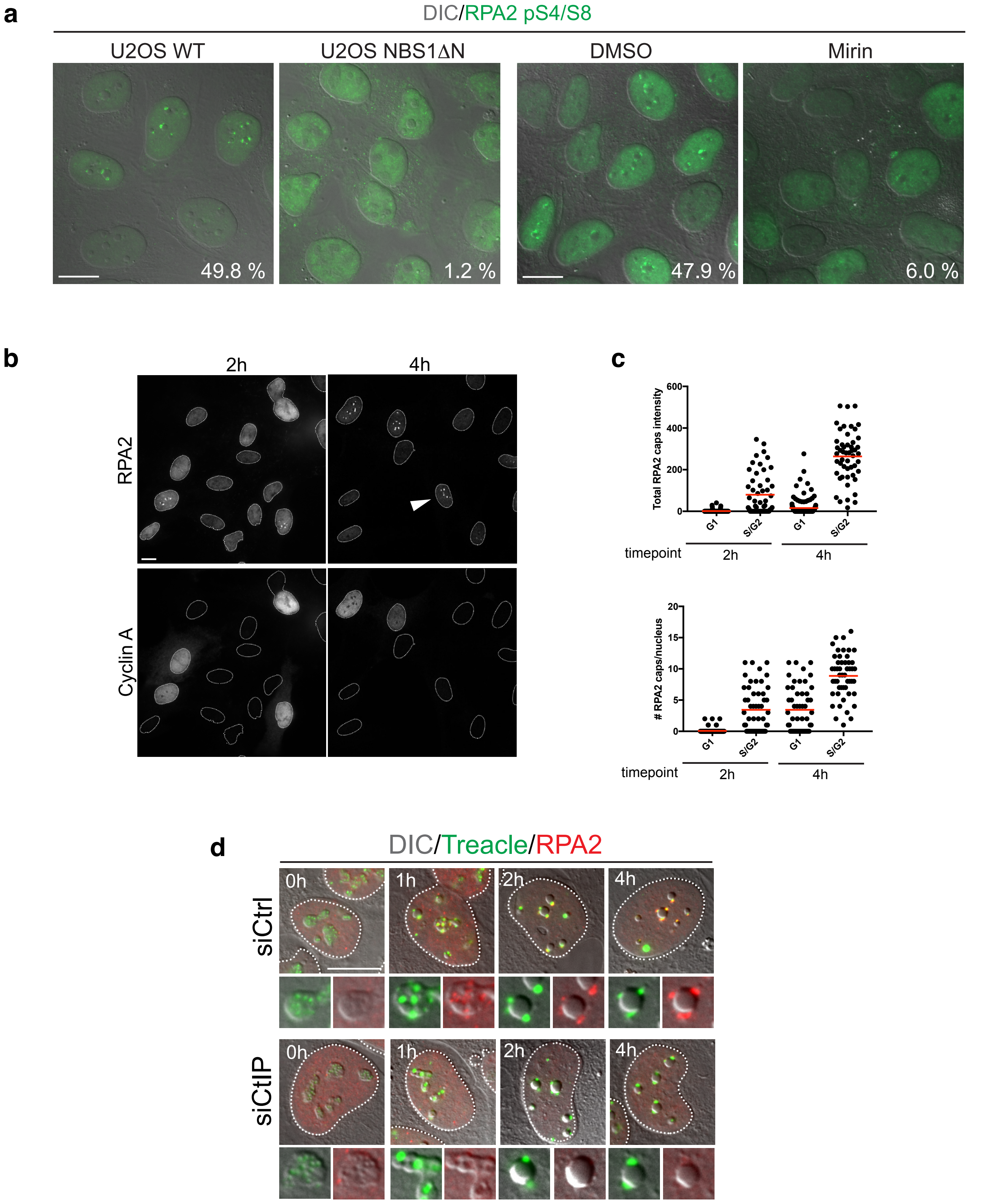

Supplementary Figure 7: Extensive resection of rDNA repeats is not required for nucleolar segregation. **a** Immunofluorescence staining with RPA pS4/8 antibody in U2OS WT and U2OS NBS1ΔN cells, as well as in U2OS WT cells treated with either DMSO or Mirin. The percentage of cells with RPA pS4/S8 signal and nucleolar caps is indicated. **b** Timecourse of RPA2 foci formation in RPE1 cells after I-Ppo1 transfection. A G1 phase cell at the 4 h timepoint with RPA2 foci in the nucleolar periphery is highlighted by an arrowhead **c** Quantification of the experiment in **b** (all data points shown, red bars represent mean). **d** Timecourse of RPA2 foci formation in CtIP depleted U2OS cells and control cells after I-Ppo1 transfection. All scalebars = 10 μm.

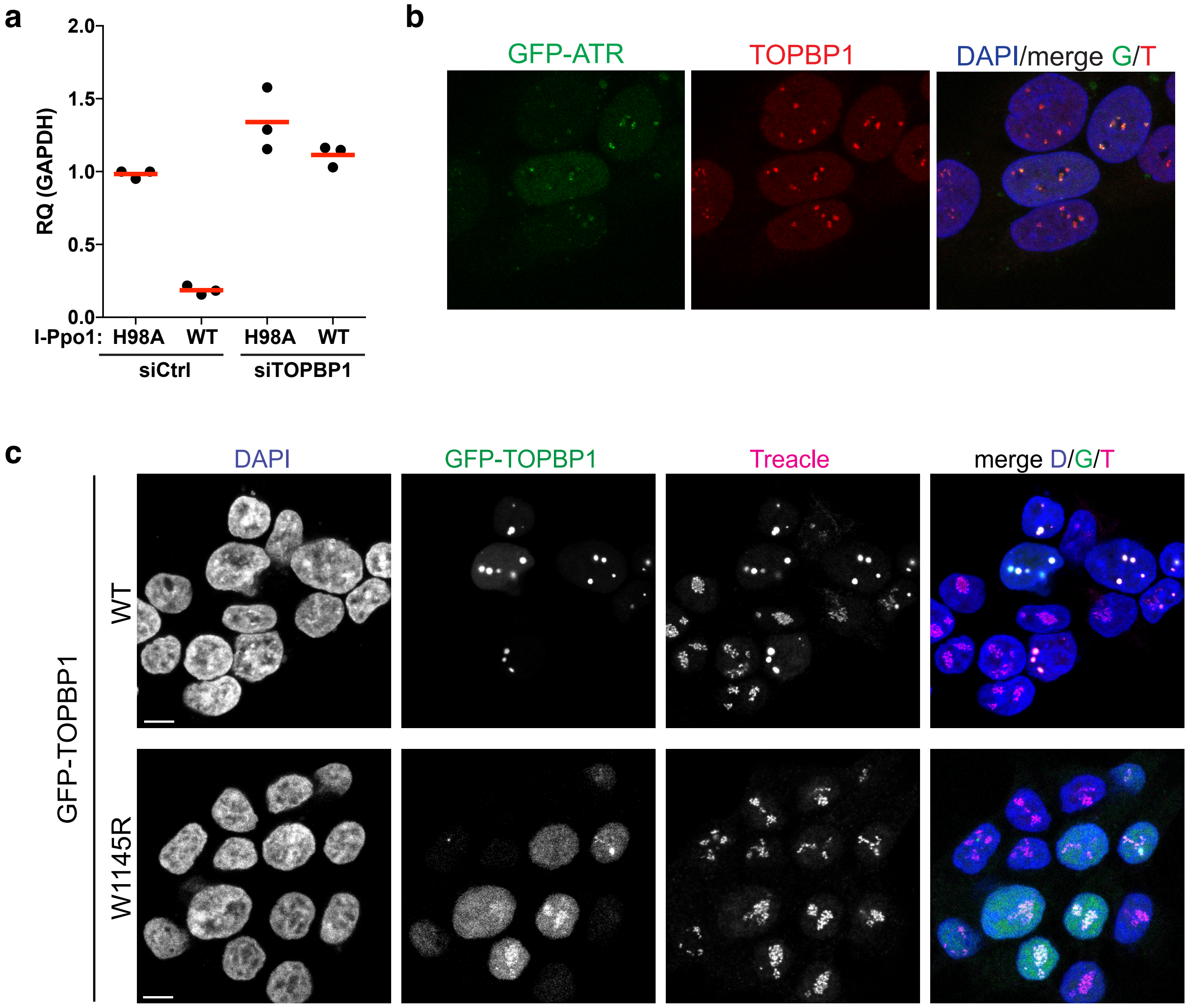

Supplementary Figure 8: Role of TOPBP1 in transcriptional repression and ATR recruitment in the nucleoli. **a** qRT-PCR of rRNA levels after I-Ppo1 transfection in control siRNA and TOPBP1 siRNA transfected U2OS cells (red bars represent mean; n=3) **b** GFP-ATR localization relative to TOPBP1 localization in GFP-ATR expressing U2OS cells 2 h ater I-Ppo1 transfection. **c** GFP-TOPBP1 and Treacle localization in GFP-TOPBP1 wild type and W1145R transfected 293FT cells. All scalebars = 10 μm.

a

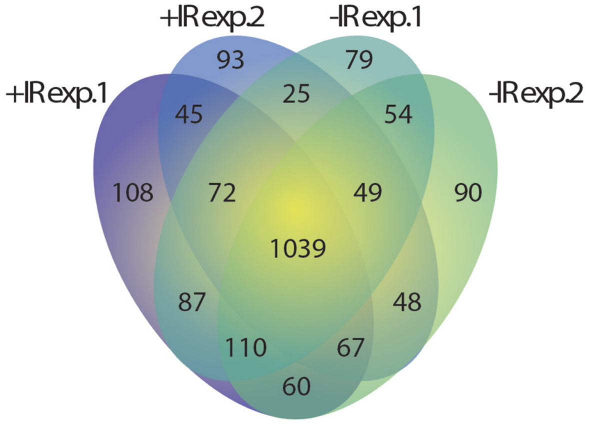

b

Protein	Intensity
TOPBP1	1.23E+11
TCOF1	3.34E+09
BLM	9.75E+08
TP53BP1	3.52E+08
RAD9A	3.14E+08
MRE11A	1.39E+08
RAD50	1.33E+08

c

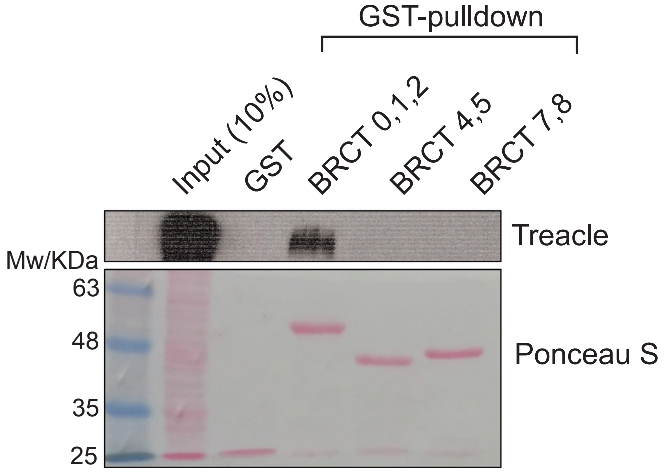

d

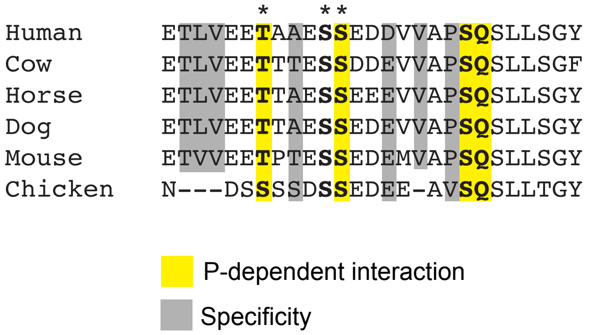

Supplementary Figure 9: Treacle-TOPBP1 interaction. **a** Summary of identified proteins in IR-treated cells and control cells (two independent experiments). **b** Average intensity of a selection of TOPBP1-associated proteins identified in all four experiments outlined in **a**. See Supplementary tabele 1 for full list of TOPBP1 associated proteins. **c** Pulldowns from HeLa nuclear extracts with purified GST-tagged TOPBP1 BRCT tandem domains. **d** Conserved putative TOPBP1 interaction motif in the C-terminal region of Treacle. * marked Ser and Thr residues: phosphorylation identified with high positional probability by mass spectrometry. Amino acid positions resembling known TOPBP1 interaction motifs are highlighted in yellow (phosphor-interaction) and grey (BRCT tandem specificity). See Supplementary tabele 2 for full list of Treacle phosphorylation sites.
