# Supplementary material for: Treacle controls the nucleolar response to rDNA breaks via TOPBP1 recruitment and ATR activation
Source: Nat Commun. 2020 Jan 8;11:123. doi: 10.1038/s41467-019-13981-x (PMC6949271; doi:10.1038/s41467-019-13981-x)
Supplement: Supplementary file 3 — Description of Additional Supplementary Files [file 41467_2019_13981_MOESM3_ESM.pdf]

## Description of Additional Supplementary Files

File Name: Supplementary Data 1
Description: List of TOPBP1-interacting proteins identified by mass spectrometry in four independent experiments. Two experiments were done using irradiated cells and two experiments were done using untreated cells. TOPBP1 (bait), TCOF1/Treacle and known TOPBP1-interacting proteins are highlighted.

File Name: Supplementary Data 2
Description: List of phosphorylation sites in Treacle identified by mass spectrometry.
The phosphorylated residues located within the TOPBP1 interacting motif (Thr1223, Ser1227 and Ser1228) are highlighted in yellow.

File Name: Supplementary Movie 1
Description: Live cell imaging of a U2OS cell expressing low levels of GFP-tagged Treacle after transfection with I-Ppo1 mRNA to illustrate morphological changes of the nucleoli in response to rDNA breaks. The numbers in the top right corner represent time after I-Ppo1 mRNA transfection in hh:mm. Scale bar = 10 µm.

File Name: Supplementary Movie 2
Description: Live cell imaging of a U2OS cell expressing NBS1-mNG after transfection with I-Ppo1 mRNA to illustrate the timing of NBS1 recruitment in the nucleoli in response to rDNA breaks. The numbers in the top right corner represent time after I-Ppo1 mRNA transfection in hh:mm. Scale bar = 10 µm.
