# Supplementary material for: Treacle controls the nucleolar response to rDNA breaks via TOPBP1 recruitment and ATR activation
Source: Nat Commun. 2020 Jan 8;11:123. doi: 10.1038/s41467-019-13981-x (PMC6949271; doi:10.1038/s41467-019-13981-x)
Supplement: Supplementary file 8 — Reporting Summary [file 41467_2019_13981_MOESM8_ESM.pdf]

nature research

Corresponding author(s): Manuel Stucki

Last updated by author(s): Dec 3, 2019

Reporting Summary

Nature Research wishes to improve the reproducibility of the work that we publish. This form provides structure for consistency and transparency in reporting. For further information on Nature Research policies, see Authors & Referees and the Editorial Policy Checklist.

Statistics

For all statistical analyses, confirm that the following items are present in the figure legend, table legend, main text, or Methods section.

n/a	Confirmed	
☐	☒	The exact sample size (*n*) for each experimental group/condition, given as a discrete number and unit of measurement
☐	☒	A statement on whether measurements were taken from distinct samples or whether the same sample was measured repeatedly
☐	☒	The statistical test(s) used AND whether they are one- or two-sided<br>*Only common tests should be described solely by name; describe more complex techniques in the Methods section.*
☒	☐	A description of all covariates tested
☒	☐	A description of any assumptions or corrections, such as tests of normality and adjustment for multiple comparisons
☐	☒	A full description of the statistical parameters including central tendency (e.g. means) or other basic estimates (e.g. regression coefficient) AND variation (e.g. standard deviation) or associated estimates of uncertainty (e.g. confidence intervals)
☒	☐	For null hypothesis testing, the test statistic (e.g. *F*, *t*, *r*) with confidence intervals, effect sizes, degrees of freedom and *P* value noted<br>*Give P values as exact values whenever suitable.*
☒	☐	For Bayesian analysis, information on the choice of priors and Markov chain Monte Carlo settings
☒	☐	For hierarchical and complex designs, identification of the appropriate level for tests and full reporting of outcomes
☒	☐	Estimates of effect sizes (e.g. Cohen's *d*, Pearson's *r*), indicating how they were calculated

Our web collection on statistics for biologists contains articles on many of the points above.

Software and code

Policy information about availability of computer code

Data collection	ZEN blue Pro 3.1 (Zeiss); LAS X (Leica Microsystems)
Data analysis	CellProfiler 3.0.0 (Broad Institute, open source); R 3.4.2 (R Development core team, open source); Fiji (ImageJ, open source); IMARIS 9.2 (Bitplane); GraphPad Prism 8 for Mac OS X; MaxQuant 1.5.3.30

For manuscripts utilizing custom algorithms or software that are central to the research but not yet described in published literature, software must be made available to editors/reviewers. We strongly encourage code deposition in a community repository (e.g. GitHub). See the Nature Research guidelines for submitting code & software for further information.

Data

Policy information about availability of data

All manuscripts must include a data availability statement. This statement should provide the following information, where applicable:

- Accession codes, unique identifiers, or web links for publicly available datasets
- A list of figures that have associated raw data
- A description of any restrictions on data availability

mass spectrometry proteomics data have been deposited to the ProteomeXchange via the PRIDE partner repository. A list of figures that have associated raw data is provided

Field-specific reporting

Please select the one below that is the best fit for your research. If you are not sure, read the appropriate sections before making your selection.

☒ Life sciences ☐ Behavioural & social sciences ☐ Ecological, evolutionary & environmental sciences

For a reference copy of the document with all sections, see nature.com/documents/nr-reporting-summary-flat.pdf

Life sciences study design

All studies must disclose on these points even when the disclosure is negative.

Sample size	No statistical method was used to predetermine sample size. All experiments were done in multiple biological replicates based on previous experiences.
Data exclusions	No data points were excluded from the analysis
Replication	All results were tested and confirmed with at least two independent experiments
Randomization	No randomization method was applied. Samples were separated in groups based on whether or not they were treated or not treated (I-Ppo1 transfection, knockdown by siRNA, etc.)
Blinding	No blinding assessment was performed.

Reporting for specific materials, systems and methods

We require information from authors about some types of materials, experimental systems and methods used in many studies. Here, indicate whether each material, system or method listed is relevant to your study. If you are not sure if a list item applies to your research, read the appropriate section before selecting a response.

Materials & experimental systems

n/a	Involved in the study	
☐	☒	Antibodies
☐	☒	Eukaryotic cell lines
☒	☐	Palaeontology
☒	☐	Animals and other organisms
☒	☐	Human research participants
☒	☐	Clinical data

Methods

n/a	Involved in the study	
☒	☐	ChIP-seq
☒	☐	Flow cytometry
☒	☐	MRI-based neuroimaging

Antibodies

Antibodies used

Target/Manufacturer/Catalogue number/Host/(clone number)/Application Immunofluorescence (IF) Western blot (WB)
BLM/Abcam/ab5446/goat/WB
GFP/Roche/11814460001/mouse/WB
HA/Santa Cruz Biotechnology/sc-57592/mouse/WB
NBS1/Abcam/ab32074/rabbit/WB
NBS1/GeneTex/GTX70224/mouse/WB
NBS1/Novus/NB100-143/rabbit/IF
TOPBP1/Bethyl Laboratories/A300-111A/rabbit/WB, IF
TOPBP1/Abcam/Ab2402/rabbit/WB
ATM pS1981/Epitomics/2152-1/rabbit/WB
ATM/Calbiochem/PC-116/rabbit/WB
ATM/Abcam/ab32420/rabbit/IF
Treacle/Sigma/HPA038237/rabbit/WB, IF
RAD9/Abcam/ab70810/rabbit/WB
RAD50/GeneTex/GTX70288/(13B3)/mouse/WB
MRE11/Abcam/ab214/(12D7)/mouse/WB
MRE11/GeneTex/GTX70212/mouse/IF
Tubulin/Sigma/DM1A/(T6199)/mouse/WB
RPA-pS4/S8/Bethyl laboratories/A700-009/rabbit/WB
RPA2 pS4/S8/Bethyl laboratories/A300-245/IF
RPA2/Abcam/ab2175/mouse/WB, IF
CHK1 pS317/Cell Signaling/2344/rabbit/WB
CHK1/Santa Cruz Biotechnology/sc-8408/mouse/WB
CHK1 pS345/Cell Signaling/2348/rabbit/WB

CHK2 pT68/Cell Signaling/2661/rabbit/WB
CHK2/Cell Signaling/2662/rabbit/WB
Nucleophosmin/Thermo Fisher/ MA5-17141/mouse/IF
BRCA1/Santa Cruz Biotechnology/sc-6454/mouse/IF
RAD51/Santa Cruz Biotechnology/sc-8349/rabbit/IF
H2AX/Millpore/05-636/mouse/IF
MDC1/Abcam/Ab50003/mouse/IF
CycA/BD biosciences/611269/mouse/IF
UBF/Santa Cruz Biotechnology/sc-13121/mouse/IF
ATR/Santa Cruz Biotechnology/sc515173/mouse/IF
V5/Abcam/ab27671/mouse/IF

Validation	Treacle (Sigma;HPA038237), validated by siRNA/WB/IF; TOPBP1 (Bethyl Laboratories), validated by siRNA/WB/IF. Other antibodies were validated by the manufacturers

Eukaryotic cell lines

Policy information about cell lines

Cell line source(s)	U2OS: Dr. Steve Jackson RPE-1: Dr. Alessandro Sartori 293-T: Dr. Michael Hottiger
Authentication	The cell lines have been authenticated based on morphological criteria.
Mycoplasma contamination	All cell lines were tested negative for mycoplasma.
Commonly misidentified lines (See ICLAC register)	No commonly misidentified cell lines were used.
